# Targeting Refractory Sarcomas and Malignant Peripheral Nerve Sheath Tumors in a Phase I/II Study of Sirolimus in Combination with Ganetespib (SARC023)

**DOI:** 10.1155/2020/5784876

**Published:** 2020-01-30

**Authors:** AeRang Kim, Yao Lu, Scott H. Okuno, Denise Reinke, Ophélia Maertens, John Perentesis, Mitali Basu, Pamela L. Wolters, Thomas De Raedt, Sant Chawla, Rashmi Chugh, Brian A. Van Tine, Geraldine O'Sullivan, Alice Chen, Karen Cichowski, Brigitte C. Widemann

**Affiliations:** ^1^Children's National Medical Center, 111 Michigan Ave., NW, Washington, DC 20010, USA; ^2^SARC Statistics, Weill Cornell Medicine Healthcare and Policy Research, 602 East 67^th^ Street, New York, NY 10065, USA; ^3^Mayo Clinic, 200 First St., SW, Rochester, MN 55905, USA; ^4^SARC, 24 Frank Lloyd Wright Drive, Ann Arbor, MI 48105, USA; ^5^Children's Hospital of Philadelphia, Univeristy of Pennsylvania, 3501 Civic Center Boulevard, 19104 Philadelphia, PA, USA; ^6^Cincinnati Children's Hospital & Uinviersity of Cincinnati, 3333 Burnet Ave., Cincinnati, OH 45229, USA; ^7^Pediatric Oncology Branch, National Cancer Institute, 10 Center Drive, Bethesda, MD 20892, USA; ^8^Sarcoma Oncology Center, 2811 Wilshire Blvd, Santa Monica, CA 90403, USA; ^9^University of Michigan, 1500 E. Medical Center Dr., SPC 5912, Ann Arbor, MI 48109, USA; ^10^Washington University in St. Louis, 660 S Euclid Ave., St. Louis, MO 63110, USA; ^11^National Cancer Institute, Developmental Therapeutics Clinic, Division of Cancer Treatment and Diagnosis, Bethesda, MD 20892, USA

## Abstract

**Purpose:**

Malignant peripheral nerve sheath tumors (MPNSTs) are aggressive soft tissue sarcomas. Combining Hsp90 inhibitors to enhance endoplasmic reticulum stress with mTOR inhibition results in dramatic MPNST shrinkage in a genetically engineered MPNST mouse model. Ganetespib is an injectable potent small molecule inhibitor of Hsp90. Sirolimus is an oral mTOR inhibitor. We sought to determine the safety, tolerability, and recommended dose of ganetespib and sirolimus in patients with refractory sarcomas and assess clinical benefits in patients with unresectable/refractory MPNSTs. *Patients and Methods*. In this multi-institutional, open-label, phase 1/2 study of ganetespib and sirolimus, patients ≥16 years with histologically confirmed refractory sarcoma (phase 1) or MPNST (phase 2) were eligible. A conventional 3 + 3 dose escalation design was used for phase 1. Pharmacokinetic and pharmacodynamic measures were evaluated. Primary objectives of phase 2 were to determine the clinical benefit rate (CBR) of this combination in MPNSTs. Patient-reported outcomes assessed pain.

**Results:**

Twenty patients were enrolled (10 per phase). Toxicities were manageable; most frequent non-DLTs were diarrhea, elevated liver transaminases, and fatigue. The recommended dose of ganetespib was 200 mg/m^2^ intravenously on days 1, 8, and 15 with sirolimus 4 mg orally once daily with day 1 loading dose of 12 mg. In phase 1, one patient with leiomyosarcoma achieved a sustained partial response. In phase 2, no responses were observed. The median number of cycles treated was 2 (1–4). Patients did not meet the criteria for clinical benefit as defined per protocol. Pain ratings decreased or were stable.

**Conclusion:**

Despite promising preclinical rationale and tolerability of the combination therapy, no responses were observed, and the study did not meet parameters for further evaluation in MPNSTs. This trial was registered with (NCT02008877).

## 1. Introduction

Malignant peripheral nerve sheath tumors (MPNSTs) are highly aggressive soft tissue sarcomas. The only known curative therapy for MPNSTs is complete surgical resection with wide negative margins [1–5], which is often not feasible due to location, size, and metastasis. Half of all MPNSTs develop in patients with neurofibromatosis type 1 (NF1), a common autosomal-dominant tumor predisposition syndrome [1, 6]. The gene responsible for NF1 encodes for the protein neurofibromin. Decreased levels of neurofibromin in NF1 lead to dysregulated Ras and tumorigenesis. NF1 loss is also seen in the majority of sporadic MPNST, suggesting NF1 is an important tumor suppressor in all MPNSTs [7]. Increased understanding in the pathogenesis of MPNSTs, availability of targeted agents, and sophisticated preclinical models have facilitated development of rational clinical trials for MPNSTs.

Mammalian target of rapamycin (mTOR) has been reported to be hyperactivated in NF1-deficient tumors as a consequence of aberrant Ras signaling [8]. Using an NF1/*p*53-mutant MPNST model, the Cichowski laboratory demonstrated that mTOR inhibitors (mTORi) suppressed tumor growth in a potent, but cytostatic manner [9] and ultimately became resistant to treatment. Identifying alternative strategies in combination with mTORi may be beneficial. Endoplasmic reticulum (ER) stress is induced when unfolded proteins accumulate in the ER [10]. Oncogenic RAS also causes ER stress [11], and when the ER stress level becomes insurmountable, cell death ensues, suggesting agents that enhance ER stress may be developed as anticancer agents. Enhancing ER stress using Hsp90 inhibitors coupled with mTORi led to tumor shrinkage in a genetically engineered MPNST mouse model, which correlated with profound damage to the ER and cell death [12]. This was only seen in tumors treated with the combination, but not in tumors exposed to either agent alone. Previously, no targeted agents have been able to cause tumor regression in a genetically engineered MPNST mouse model or human MPSNT trials.

Ganetespib is a novel injectable potent small molecule inhibitor of Hsp90. It has a favorable safety profile, including minimal ocular toxicity, and promising antitumor activity in a broad-range tumor type [13]. Sirolimus is an oral commercially available mTORi with a long safety record and demonstrated efficacy in cancer models [14–16]. Preclinical data to support this combination in other bone and soft tissue sarcomas provided rationale to include all sarcomas in the phase 1 component [17–21]. Based on strong preclinical rationale, we sought to determine whether the combination of ganetespib with sirolimus will be safe, tolerable, and cause tumor regression in patients with refractory MPNSTs.

## 2. Materials and Methods

### 2.1. Patient Population

Patients aged ≥16 years with histologically confirmed unresectable/refractory sarcoma (phase 1) and MPNST (phase 2) with measurable disease per WHO criteria [22]; Eastern Cooperative Oncology Group performance status of 0 to 2; adequate bone marrow, liver, and renal function; fasting serum cholesterol and triglycerides ≤300 mg/dL; and QTcF ≤480 ms were eligible. Patients had recovered from all prior therapy. For patients with NF1, diagnostic criteria for NF1 were documented [23].

The multi-institutional trial was coordinated through Sarcoma Alliance for Research through Collaboration (SARC) funded by the Department of Defense Clinical Trial Award. Ganetespib was supplied by Synta Pharmaceuticals, and sirolimus was purchased commercially and provided through the study. The study was conducted after approval from institutional review boards from all participating sites, and all patients provided written informed consent before participating.

### 2.2. Study Design

Phase 1 was a standard 3 + 3 dose escalation study to determine the maximum tolerated dose (MTD) or recommended phase 2 dose (RP2D) of ganetespib with sirolimus. In the absence of dose-limiting toxicities (DLTs), three patients were to be treated in each dose cohort. DLTs were defined as grade 4 hematological toxicity, any grade ≥3 nonhematological toxicity with the exception of grade 3 nausea and vomiting of <3 days duration, grade 3 diarrhea ≤3 days duration, grade 3 alanine aminotransferase (ALT)/aspartate aminotransferase (AST) that returned to ≤grade 1 within 7 days of study drug interruption, grade 3 fever or infection <5 days, and any grade 3 electrolyte imbalances that responded to oral or intravenous supplementation. Any grade 2 nonhematological toxicity that persisted for ≥7 days and is considered medically significant or intolerable by patients and any adverse event requiring interruption of study drug for ≥7 days or which recurred upon drug challenge was also dose limiting. Toxicity was graded according to National Cancer Institute Common Terminology Criteria for Adverse Events (CTCAE) (version 4.0). The MTD was defined as the dose level immediately below the dose at which ≥33% of patients in a cohort experience a DLT in first treatment cycle. A patient was considered evaluable for MTD if at least 85% of prescribed sirolimus dose was given unless held for toxicity.

Ganetespib was administered intravenously (IV) over one hour on days 1, 8, and 15 of each 28-day cycle, and sirolimus administered orally once daily continuously after day 1 loading dose. Phase 1 dose levels are summarized in Table 1. Only one planned dose escalation with ganetespib at 200 mg/m^2^ was planned. There were no plans to escalate beyond the single-agent recommended doses of either agent. Patients on phase 2 were all treated at the RP2D. The primary objective of the phase 2 trial was to determine the clinical benefit rate (CBR) of ganetespib in combination with sirolimus for patients with MPNSTs defined as CR, PR, or SD ≥4 months using WHO criteria of ganetespib in combination with sirolimus for patients with MPNSTs. Secondary objectives assessed changes in pharmacodynamic parameters in blood and pain using patient-reported outcomes (PROs).

### 2.3. Assessments

Patients were evaluated with weekly history and physical and laboratory assessments during cycle 1 and then prior to each cycle with laboratory assessments performed every other week during subsequent cycles. EKG was performed at baseline; cycle 1, day 2; and then prior to every odd cycle. Radiographic disease evaluation for tumor evaluation was performed at baseline and prior to odd cycles. Patients who experienced disease progression based on WHO criteria but felt to be receiving benefit per treating investigator were allowed to continue on treatment as long as they had stable disease per RECIST 1.1 criteria [24] and had not met any other off treatment criteria.

PROs assessing pain and pain interference using two validated scales were performed at baseline and then prior to every odd cycle. The Numerical Rating Scale-11 (NRS-11) [25] assessed pain severity from 0 to 10 (4–6 = moderate pain; 7–10 = severe pain), and the Brief Pain Inventory (BPI) [26] assessed the impact of pain on daily activities for which the total score is the mean of the seven items rated 0–10. Clinically, meaningful change is ≥2 and ≥1 point, respectively [27, 28].

### 2.4. Pharmacodynamics (PD)

Changes in PD markers in peripheral blood mononuclear cells were performed prior to treatment and cycle 1, day 15, 6 hours after ganetespib administration. Western blot analyses were performed for Akt, phospho(*p*)-Akt, eiF2*α*, *p*-eIF2*α*, *p*-S6, and Hsp70. Signal intensity of the autoradiogram was quantified using densitometry scanning and analyzed using ImageJ software (National Institutes of Health, Bethesda, MD). The absorbance of each phosphoprotein lane was recorded, and protein levels were determined after normalizing for levels of corresponding total protein. Histone H3 was used as a protein loading control.

### 2.5. Pharmacokinetics

Pharmacokinetics were required for all patients treated on phase 1 portion and optional for phase 2 patients. Blood samples (3 mL) each were collected on day 1 prior to treatment, and then on cycle 1, day 15 to capture steady-state sirolimus levels. Ganetespib samples were collected at hours 0, 1 (end of infusion), 2, 4, 6, 8, and 24. Sirolimus samples were collected at hours 0, 1, 2, 4, and 24. Pharmacokinetic samples for ganetespib were evaluated at Synta Pharmaceuticals and at Cincinnati Children's Hospital for sirolimus. Pharmacokinetic analysis was conducted using noncompartmental methods using Phoenix® WinNonlin version 6.2.1 software.

### 2.6. Statistical Methods

Descriptive data are reported as frequencies, proportions, means, medians, and ranges. An evaluable patient was classified a responder (success) for the primary endpoint if the patient achieves a PR, CR, or stable disease at ≥4 months as defined by the WHO criteria. The target CBR was 25%, and a CBR ≤5% was considered uninteresting. Using Simon's optimal two-stage phase II design, the first stage required 10 patients, with no further accrual if 0 of 10 patients respond. If ≥1/10 patients respond, accrual would continue until a total of 20 patients have been enrolled. If ≥3/20 patients respond, this combination would be considered to have sufficient activity. Assuming the number of successes is binomially distributed, this design has a one-sided alpha of 0.07 and a power of 88% for detecting a true success probability of at least 25% versus the null hypothesis success rate of 5% or less.

PD endpoints were analyzed using the GraphPad prism 6.0 statistical software. *p* values were calculated using a two-way analysis of variance. A *p* value of <0.05 was considered to indicate a statistically significant result. Data were normalized to highest value within each patient group.

Due to the small number of patients that reached their follow-up, PRO evaluations due to progressive disease, changes in individual pain scores, and the mean overall changes from baseline to their last PRO evaluation are described.

## 3. Results

Twenty patients were enrolled, 10 in each phase. The baseline characteristics are listed in Table 2. A heterogeneous population of sarcomas enrolled on phase 1, including 3 patients with NF1-associated MPNSTs. The majority of patients had metastatic disease (90%) and prior therapy including surgery, chemotherapy, and radiation therapy. In phase 2, half had NF1-associated MPNST.

### 3.1. Determining RP2D

There were no DLTs (*n*=3) on the first dose level. At dose level 2 (*n*=6, 1 patient unevaluable), one patient of the first three enrolled in this cohort had a DLT of grade 4 thrombocytopenia. Three additional patients enrolled onto this cohort without additional DLTs to confirm the R2PD of ganetespib 200 mg/m^2^ IV on days 1, 8, and 15 with sirolimus 4 mg orally once daily continuous with a cycle 1 day 1 loading dose of 12 mg. All patients in phase 2 were treated at the RP2D.

### 3.2. Toxicities

Grade ≥3 toxicities are listed in Table 3. Toxicities were manageable, and the most frequent non-DLT toxicities were diarrhea, elevated liver transaminases, and fatigue. No significant visual or cardiac toxicities were associated with either agent. Grades 1 and 2 ganetespib-related infusion reactions were observed (*n*=4), but all patients were successfully managed with diphenhydramine and steroids, and all patients received subsequent doses without dose reduction. All Phase 2 patients were required to receive premedication with diphenhydramine and steroids prior to ganetespib infusion. Two patients in phase 2 were removed from therapy due to toxicity. The first was due to grade 4 AST which did not recover within time frame required by protocol, and the other exhibited grade 3 dehydration which recovered, but subsequently developed elevated bilirubin while off treatment, and did not meet parameters to restart therapy within time frame required by protocol.

### 3.3. Pharmacokinetics

All but one patient participated in pharmacokinetics in phase 1. One patient in DL2 was not included in analysis due to dose reduction prior to collection. Due to sampling time inconsistencies and assay sensitivity, most pharmacokinetic parameters (AUC, *C*_max_, Vss, clearance) could not be reliably determined. The mean (SD) sirolimus trough in dose level 1 (*n*=2) was 12.1 (3.6) ng/mL and 12.5 (8.9) ng/mL in dose level 2 (*n*=5). These ranges are typically considered therapeutic for sirolimus [14]. The mean (SD) ganetespib *t*_1/2_ was 6.4 (2.1) hours and 6.0 (0.9) hours. Although complete pharmacokinetic parameters were not able to be determined, the levels for sirolimus and half-life of ganetespib were consistent with previous single agent and combined study findings [29, 30].

### 3.4. Patient-Reported Outcomes

Thirteen subjects in the phase 1 and 2 cohorts combined had MPNSTs and completed the prestudy pain evaluation; 10 out of 13 (77%) rated having some degree of pain. At baseline, the mean (SD, range) overall pain intensity, tumor pain intensity, and pain interference score were 4.8 (3.9; 0–10), 5.1 (3.8, 0–10), and 3.9 (3.0, 0–10), respectively. Four of the 13 subjects completed both the baseline and pre-cycle 3 evaluation (one reached pre-cycle 5). The other 9 subjects were taken off study due to disease progression (*n*=7), toxicity (*n*=1), and death (*n*=1) prior to reaching cycle 3. In this small cohort, we observed clinically meaningful improvement in overall and tumor pain intensity and pain interference score from baseline to either pre-cycle 3 or 5 evaluation with a mean difference of 2.75, 2.5, and 3.35, respectively (Tables 4 and 5). All patients had progressive disease.

### 3.5. Response

In phase 1, patients received a median of 2 cycles (range, 2–34). One patient had a confirmed, sustained partial response and came off study at cycle 34 due to investigator choice. This patient with a history of progressive painful leiomyosarcoma also had a dramatic clinical response with improved pain and function. Among the ten patients enrolled on the first stage of phase 2, none achieved clinical benefit as defined by the protocol. The study did not demonstrate activity sufficient to open Stage 2. The median number of cycles treated was 2 (1–4). One patient considered a nonresponder by the WHO criteria after 4 cycles of therapy. The patient had three target lesions with two of the targets measuring stable disease and one target demonstrating ≥25% increase in area, thus meeting the definition of progression per the WHO. The patient had stable disease per RECIST criteria when evaluating the sum of the largest dimension in all three targets, and the treating physician felt the patient was receiving benefit through slowed progression. The study was amended to allow patient to continue with treatment until progression per RECIST. The patient continued until progression per RECIST after 8 cycles of therapy. Of note, this patient also had clinically significant decrease in tumor pain intensity (−3) and BPI (−3.57) from baseline. Three patients with MPNST had shrinkage in some targets but growth in other target lesions leading to an overall PD by the WHO.

### 3.6. Pharmacodynamics

**Figure 1:**
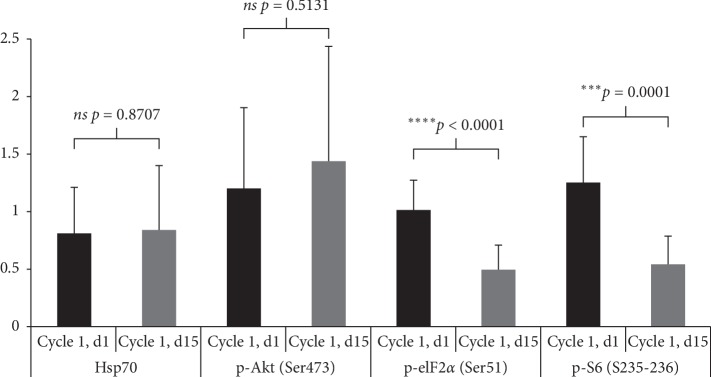
Aggregate pharmacodynamic responses to ganetespib and sirolimus therapy.

The effects of the combination of sirolimus and ganetespib on biomarkers of Hsp90 and mTOR pathway inhibition were examined in PBMC samples of 11 patients who provided consent and had adequate specimens for analysis (Figure 1). We observed consistent inhibition of *p*-S6 (read-out for mTOR pathway inhibition) by day 15 in all patients (mean 56% inhibition; range 25–79%). *p*-Akt levels varied significantly at steady state, ranging from over 4-fold increase to 83% inhibition. Though associated with a negative feedback loop for sirolimus mTOR inhibition, the changes in *p*-Akt could not be correlated with response or toxicity. We observed consistent and statistically significant inhibition of *p*-eIF2*α* (read-out of UPR activation) in all samples. Overall levels of Hsp70 (read-out of Hsp90 inhibition) varied greatly with six patients exhibiting a 91% increase at day 15, two with minimal change, and three with significant decrease. These changes could not be correlated with response or toxicity due to small numbers.

## 4. Discussion

We established an RP2D of 200 mg/m^2^/dose of ganetespib IV on days 1, 8, and 15 with sirolimus 4 mg orally continuously (with day 1 loading dose of 12 mg) for a 28-day cycle. This is the RP2D for both agents as monotherapy, suggesting that the combination does not have intolerable overlapping toxicities. Although complete pharmacokinetic parameters were unable to be fully determined, sirolimus trough levels were within expected therapeutic levels and the half-life of ganetespib was consistent with previous studies. Sirolimus does not appear to influence ganetespib pharmacokinetics. There was consistent inhibition of *p*-S6 in PBMC at steady state, reflective of likely effective therapeutic exposure to sirolimus. Upregulation of Hsp70, a putative biomarker of Hsp90 inhibition, varied greatly between patients, consistent with previous studies of ganetespib [29, 31]. The most common adverse event seen was diarrhea, which was manageable with loperamide therapy. Infusion-related reactions with ganetespib were frequent but manageable with premedications.

This multi-institutional SARC coordinated study was successful in terms of study implementation for a very rare disease. Phase 1 was completed in a timely manner with limited patients. The initial stage of phase 2 fully accrued in 6 months. Unfortunately, our study did not meet the required parameters to open the second stage, and this combination was determined to have insufficient activity in MPNST to move forward.

To date, clinical trials with noncytotoxic targeted therapy have yet to demonstrate an objective response in MPNST using traditional radiographic measurements such as RECIST or WHO [32]. The outcome measurement used to determine the response in this study was CBR using the WHO criteria [22]. These criteria were used because MPNSTs are typically complex nonspherical tumors, and bidimensional measurements may reflect better changes in tumor size, but mainly, it was selected to allow for a consistent comparison with previous phase 2 trials of MPNSTs, which also used the WHO criteria. The WHO criteria define progression as *a* ≥25% increase in one or more measurable lesions or appearance of new lesions. Thus, it is possible that the sum of the products decreases, but a patient meets criteria for progression based on an increase in just one lesion. Several patients demonstrated heterogeneous responses radiographically, and many had symptomatic improvement in pain as demonstrated by the clinically meaningful changes in PRO pain scores. The more stringent criteria may put finding any signal of interest for further pursuit in this disease at a higher standard than other phase 2 trials which primarily use RECIST for activity. Which standard response measurements are optimal for primary outcome of novel agents in this patient population is not known. Thus, other outcome measurements should be evaluated and incorporated into clinical trials such as PROs and functional imaging such as FDG-PET or magnetic resonance imaging apparent diffusion coefficient. These types of imaging biomarkers are being used more frequently in assessment of response in sarcomas and appear to be better correlated with histologic response than 1D or 2D measurements [33–36].

Our highly refractory pretreated population may affect tumor response and microenvironment. PD surrogate blood markers in this small sample set demonstrated consistent mTOR inhibition, but changes in *p*-Akt were highly variable. Although inconsistent in terms of up- or downregulation in our samples, *p*-Akt is typically considered a negative feedback loop for sirolimus mTOR inhibition and may have also contributed to the lack of responses. Changes in Hsp70 were also highly variable, and the study may not have achieved biologically effective levels of ganetespib, although increasing the dose would unlikely have been tolerable. Significant challenges remain with the direct measurement of Hsp90 inhibition, and unknown mechanisms may have also interfered with effect. To better understand target inhibition and mechanisms of resistance that differ among patients and mouse models, tumor tissue and surrogate markers should be collected and evaluated and include additional client proteins that may be more informative. Attempts should be made to collect PROs and PD markers in all patients and at earlier time points, as inconsistent sampling will not allow to draw meaningful conclusions.

Overall, patients were able to tolerate the combination therapy with HSP90 and mTOR inhibition. We were able to determine a recommended dose of this combination therapy. However, no responses were observed, and the study did not meet parameters for further evaluation in MPNSTs.

## 5. Conclusions

Despite promising preclinical rationale and tolerability of the combination therapy, no responses were observed, and the study did not meet parameters for further evaluation of this combination in this population. This trial was successful in rapid accrual and execution and gave insight into future design and development of targeted therapy for MPNST. Further efforts to rapidly develop and translate the most promising therapies in this aggressive sarcoma are ongoing.

## Figures and Tables

**Table 1:** Phase 1 dose escalation schema.

Dose level	Ganetespib (mg/m^2^), intravenously on days 1, 8, and 15 of each 28-day cycle	Sirolimus (mg), oral		Number of evaluable patients
		Loading dose, cycle 1, day 1 only	Maintenance dose, orally once daily continuously	
−2	100	6	2	–
−1	150	6	2	–
1^*∗*^	150	12	4	3
2	200	12	4	6

^*∗*^Starting dose: 1 cycle = 28 days.

**Table 2:** Baseline patient characteristics.

Characteristic	Phase 1 (*n*=10)	Phase 2 (*n*=10)
Median age, years (range)	26 (16–89)	38 (24–61)
Female, *n* (%)	2 (20)	4 (40)

Sarcoma subtype, *n*		
Alveolar soft part sarcoma	1	
Ewing sarcoma	1	
Leiomyosarcoma	2	
Liposarcoma	3	
MPNST	3	10
*NF1 associated, n (%)*	**3 (100)**	**5 (50)**
*Sporadic, n (%)*	**0 (0)**	**5 (50)**

Tumor location at diagnosis, *n*		
Abdomen	1	1
Extremity	1	4
Head	1	0
Lung	0	1
Mediastinum	1	0
Peritoneum	1	0
Skin	1	0
Spine	1	2
Other	3	2

Primary tumor resected, *n* (%)	8 (80)	4 (40)
If yes, margins		
R0: microscopic negative	1 (12.5)	2 (50)
R1: microscopic positive	1 (12.5)	0 (0)
R2: gross residual disease	1 (12.5)	0 (0)
Unknown	5 (62.5)	2 (50)

History of metastatic disease, *n* (%)	9 (90)	9 (90)
Prior chemotherapy regimen, *n* (%)	10 (100)	8 (80)
Prior radiation, *n* (%)	7 (70)	6 (60)
Prior surgery, *n* (%)	10 (100)	9 (90)

**Table 3:** The combined phase 1/2 grade ≥3 toxicities separated by attribution.

	All grade 3	All grade 4	Related grade 3	Related grade 4
Blood lymphatic				
Lymphocyte count decreased	3 (15)	1 (5)	2 (10)	1 (5)
Platelet count decreased	1 (5)	1 (5)	1 (5)	1 (5)
White blood cell decreased	1 (5)			

Gastrointestinal				
Abdominal pain	1 (5)			
Diarrhea	3 (15)		3 (15)	
Nausea	1 (5)			
Obstruction gastric	1 (5)			
Vomiting	1 (5)			

General				
Edema limbs	1 (5)			
Fever	1 (5)			
General disorders and administration site conditions—other, specify	1 (5)			

Hepatobiliary disorders				
Cholecystitis	2 (10)		1 (5)	
Infections and infestations				
Lung infection	1 (5)			
Investigations				
Alanine aminotransferase increased	1 (5)		1 (5)	
Alkaline phosphatase increased	3 (15)		2 (10)	
Aspartate aminotransferase increased		1 (5)		1 (5)

Metabolism and nutrition				
Dehydration	2 (10)		1 (5)	
Hypercalcemia	1 (5)		1 (5)	
Hyperglycemia	1 (5)			
Hypertension	1 (5)		1 (5)	
Hypoglycemia	1 (5)			
Hypokalemia	1 (5)		1 (5)	
Hyponatremia	1 (5)			

Musculoskeletal and connective tissue disorders				
Back pain	1 (5)			
Neoplasms benign, malignant, and unspecified				
Tumor pain	1 (5)			

**Table 4:** NRS-11 ratings of pain intensity from baseline to pre-cycle 3/5.

Patient	Tumor pain			Overall pain		
	Baseline	PC3/5	Diff	Baseline	PC3/5	Diff
009	10	10	0	10	10	0
013	5	2^†^	−3	5	1^*∗*^	−4
016	10	8	−2	10	8	−2
019	5	0	−5	5	0	−5
Mean	7.5	5	2.5	7.5	4.75	2.75

^*∗*^Ratings from the pre-cycle 5 evaluation. *Note*. Clinically meaningful change is ≥2 points (0–3 = mild pain; 4–6 = moderate pain; 7–10 = severe pain).

**Table 5:** BPI ratings of pain interference from baseline to pre-cycle 3/5.

Patient	Adults (*n*=4)		
	Baseline	PC3/5	Diff
009	5.29	5.71	0.42
013	4.43	0.86^*∗*^	−3.57
016	9.43	6.0	−3.43
019	6.8	0	−6.8
Mean	6.49	3.14	3.35

^*∗*^Ratings from the pre-cycle 5 evaluation. *Note*. Clinically meaningful change is ≥1 point.

## Data Availability

The data used to support the findings of this study are available from the corresponding author upon request.
